# MHiC, an integrated user-friendly tool for the identification and visualization of significant interactions in Hi-C data

**DOI:** 10.1186/s12864-020-6636-7

**Published:** 2020-03-12

**Authors:** Saman Khakmardan, Mohsen Rezvani, Ali Akbar Pouyan, Mansoor Fateh, Hamid Alinejad-Rokny

**Affiliations:** 1 Faculty of Computer Engineering, Shahrood University of Technology, Shahrood, Iran; 2 Systems Biology and Health Data Analytics Lab, The Graduate School of Biomedical Engineering, UNSW Sydney, Sydney, 2052 Australia; 3 School of Computer Science and Engineering, The University of New South Wales (UNSW Sydney), Sydney, 2052 Australia

**Keywords:** Chromosome conformation capture, Hi-C, Statistically significant interactions, Hi-C data visualization, Contact map

## Abstract

**Background:**

Hi-C is a molecular biology technique to understand the genome spatial structure. However, data obtained from Hi-C experiments is biased. Therefore, several methods have been developed to model Hi-C data and identify significant interactions. Each method receives its own Hi-C data structure and only work on specific operating systems.

**Results:**

We introduce MHiC (Multi-function Hi-C data analysis tool), a tool to identify and visualize statistically signifiant interactions from Hi-C data. The MHiC tool (i) works on different operating systems, (ii) accepts various Hi-C data structures from different Hi-C analysis tools such as HiCUP or HiC-Pro, (iii) identify significant Hi-C interactions with GOTHiC, HiCNorm and Fit-Hi-C methods and (iv) visualizes interactions in Arc or Heatmap diagram. MHiC is an open-source tool which is freely available for download on https://github.com/MHi-C.

**Conclusions:**

MHiC is an integrated tool for the analysis of high-throughput chromosome conformation capture (Hi-C) data.

## Background

Chromosome conformation capture (3C) assays are now the method of choice to study the role of DNA looping in transcriptional regulation. These assays directly identify genomic loci that are in close enough proximity to each other in living cells to be cross-linked. This new technology allows for the mapping of chromatin interactions on a whole genome level. The first study of 3C technology was developed by Dekker et al. [1]. This protocol captures interactions between a single pair of candidate regions. The other protocols include 4C (chromosome conformation capture-on-chip) which captures interactions between one locus and all other genomic loci [2], 5C (chromosome conformation capture carbon copy) which captures interactions between all locus within a given region [3], and Hi-C which captures all vs all interactions across the genome [4]. Hi-C is a high-throughput technique to understand the spatial organization of chromosomes by finding all of the nuclear interactions. Capture based methods are also developed to use biotinylated RNA oligomers complementary to enrich 3C and Hi-C libraries for specific loci of interest. These methods include Capture-C, Capture-3C, and Capture Hi-C.

The central goal in the analysis of Hi-C data is to understand which pair of genomic loci tends to interact together. Unfortunately, due to the Hi-C protocol and process, data obtained from Hi-C is biased. Therefore, normalization of Hi-C data and the identification of true interactions compared to artefact interactions is important before any downstream analysis. In Hi-C data, there are different sources of bias. The source of some types of these biases are known. For instance, spurious self-ligated interactions and PCR duplicates are easily handled at the start of Hi-C data processing from Hi-C raw data. In contrast, there are some unknown sources of bias which cannot be identified directly, and only their effect on some features can be identified. An example is ligations between two noncrosslinked DNA fragments. These interactions are indistinguishable from real interactions.

Several methods have been developed to deal with the biases such as GOTHiC [5], HiCNorm [6], and Fit-Hi-C [7]. GOTHiC is a method proposed by Mifsud et al. It uses cumulative binomial tests to identify significant interactions between distal genomic loci that have significantly more reads than expected by chance in Hi-C experiments. It can be used for both Hi-C and capture Hi-C experiments. HiCNorm models biases at lower resolutions and uses Poisson regression to normalize read counts between two-locus pair. Another method, Fit-Hi-C uses the binomial distribution to model these interactions. This method modifies the binning procedure with a two-step spline-fitting procedure. This method replaces the binning procedure with a spline-fitting procedure.

One of the main issues with these methods are that they accept a contact map in a very strict format. In other words, users need to convert the Hi-C contact map generated by Hi-C data analysis tools such as HiCUP [8] or HiC-Pro [9] to a specific format that is accepted by each background model.

**Fig. 1:**
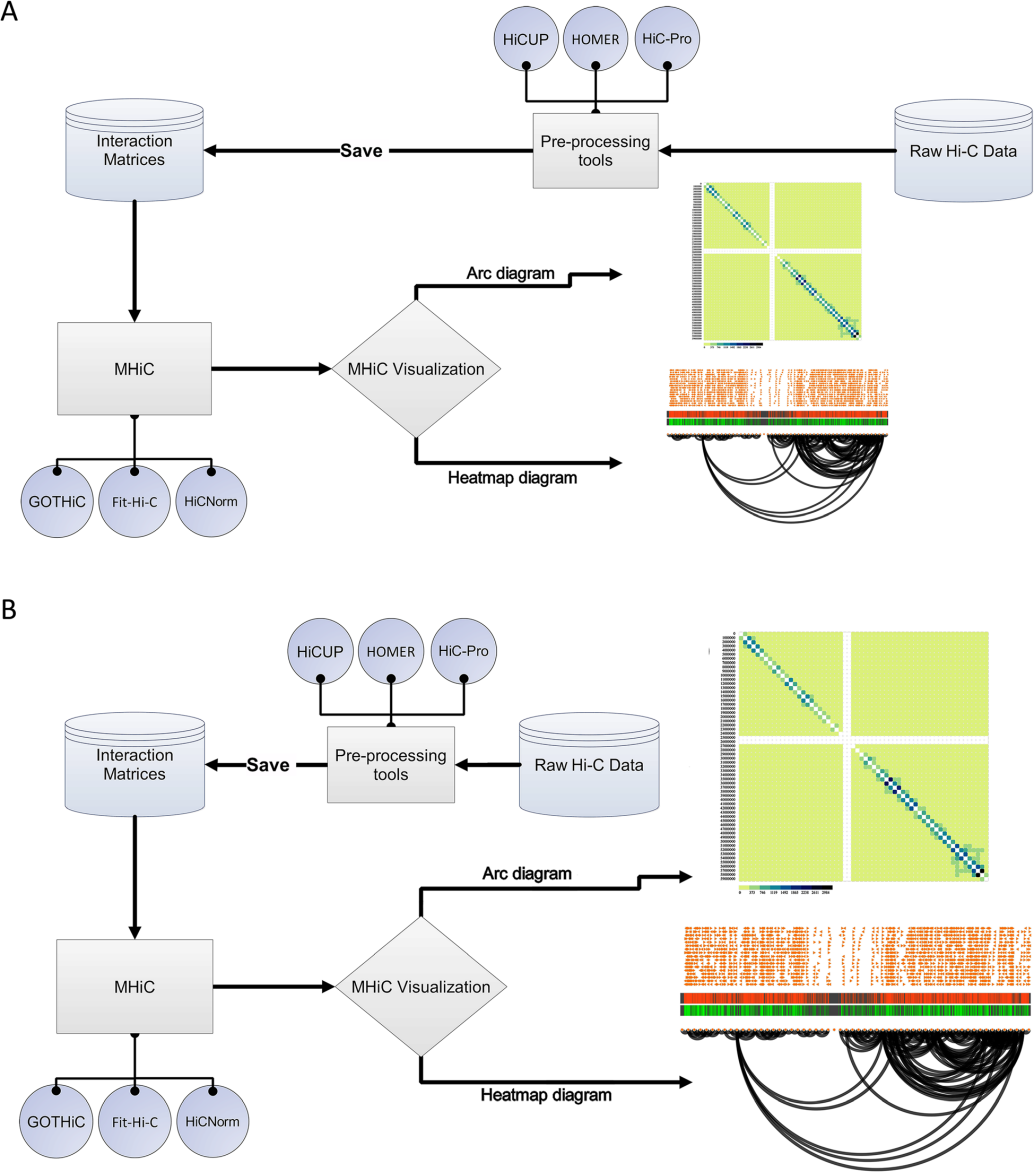
MHiC overview flowchart

In order to address the above mentioned challenges in Hi-C tools, we have developed an integrated tool called “MHiC” (Multi-function Hi-C data analysis software), which uses GOTHiC, HiCNorm and Fit-Hi-C methods with a graphical user interface (GUI) to identify statistically significant interactions in Hi-C contact maps generated by different Hi-C analysis tool. MHiC accepts HiCUP [8], HiC-Pro [9] and HOMER [10] outputs which are used to analyze raw Hi-C data and generate a Hi-C contact map, as shown in Fig. 1. MHiC also offers a flexible visualization interface to visualize raw Hi-C contact map or statistically significant interactions in both an Arc diagram and a standard Hi-C contact map (Heatmap diagram). Arc diagrams use circular nodes to show locus positions. For each interaction, an Arc link is drawn between two nodes.

In the next sections, we describe the implemented background models in MHiC and the visualization part (Additional files 1 and 2). We applied MHiC on a mouse embryonic stem cell sample from the Dixon database [11].

## Method and materials

### Input data for MHiC

MHiC accepts contact maps (Hi-C interactions) on three different formats generated by leading Hi-C analysis tools HiCUP, HiC-Pro, and Homer. MHiC accepts contact maps (Hi-C interactions) on three different formats generated by leading Hi-C analysis tools HiCUP, HiC-Pro, and Homer. After getting contact maps from these tools, MHiC converts it to a single matrix with at least 5 columns: id, fragment 1 chromosome, fragment 2 chromosome, fragment 1 start position, and fragment 2 start position (for HiCNorm method this matrix has 8 columns including GC content, effective length, and mappability features). Then, MHiC does some preprocess on data; such as changing the data resolution, calculating mid locus positions or removing diagonal interactions. In the next step, data format changes to GOTHiC, HiCNorm, or Fit-Hi-C background models formats based on user needs. In the final step, MHiC store and visualize the result from the modeling result.

### HiCUP

HiCUP [8] is a pipeline produced by the Babraham Institute to map and perform quality control on Hi-C data. HiCUP outputs include two text files. The first is a file with four columns: id, flag, chromosome and locus position. The second is a digest file which includes chromosome ID, fragment start position and fragment end position. In the first file, two separate rows with the same ID define an interaction. In order to create this structure, users should use the hicup2gothic script, which is available as a HiCUP tool.

### HiC-pro

HiC-Pro [9] is developed by Nicolas Servant to process Hi-C data from raw FASTQ files into the normalized contact maps. The HiC-Pro output is a matrix file with three columns: Locus1 ID, Locus2 ID and Interaction counts (number of interacting read between two locus), and a bed file with four columns: chromosome ID, fragment start position, fragment end position, and fragment ID.

### Homer

HOMER [10] is an analysis tool that contains several programs and analysis routines to facilitate the analysis of Hi-C data. In the Hi-C data processing section, HOMER process FASTQ and bowtie2 files to map and perform quality control on Hi-C data. In this process, HOMER creates some CSV files to define Hi-C interactions for the next processing steps. In order to create this structure, users should visit the HOMER website (http://homer.ucsd.edu).

To identify Hi-C significant interactions and visualize Hi-C contact maps, we have developed MHiC in two main modules. The first module of MHiC is implemented as an R package to provide multiple backgrounds and correction models. The second module is a user-friendly graphical interface, which provides an interactive environment for users to plot Hi-C interactions in both an Arc diagram and a contact map diagram. MHiC accepts input data from different tools such as HiCUP, HiC-Pro and HOMER and then identifies significant interactions through the GOTHiC, HiCNorm and Fit-Hi-C methods at a desired resolution of the contact map.

### Identifying significant interaction with MHiC

We developed MHiC based on the GOTHiC, HiCNorm, and Fit-Hi-C background models. These methods use different mathematical models to identify significant interactions. In the following, we explain each of the models in detail.

### GOTHiC

GOTHiC was developed by Mifsud et al. This method assumes both ends of each read-pair are affected by biases. Therefore, the probability of observing *n*_*j*, *h*_ or more read-pairs between two loci, j and h, by chance in a dataset of N reads is given by the cumulative binomial density:

1 $$ pva{l}_{j,h}=1-{\sum}_{i=0}^{n_{j,h}-1}\left(\underset{i}{N}\right){\left({p}_{j,h}\right)}^i\left(1-{p}_{j,h}\right){N}^{-i} $$

where the probability that a read pair is the consequence of a spurious ligation between two sites is:

2 $$ {p}_{j,h}=2\ast relativecoverag{e}_j\ast relativecoverag{e}_h $$

Immediately following eq. 2, the relative coverage of a given site or region is:

3 $$ relativecoverag{e}_j=\frac{reads_j}{2N} $$

where *reads*_*j*_ is the mapped read count for genomic locus_j_. After calculating the probabilities, this method uses the Benjamini-Hochberg multiple-testing correction to obtain a false discovery rate adjusted *p*-value (q-value), which is used to find significant interactions. The Benjamini-Hochberg Procedure is a technique that decreases the false discovery rate. Adjusting the rate helps to control the fact that sometimes small *p*-values (less than 5%) happen by chance, which could lead you to incorrectly reject the true null hypotheses. In this method, the p-values are first sorted and ranked. Then, each p-value is multiplied by m, the number of comparisons, and divided by its assigned rank, r_j, h_, to give the adjusted p-values.

4 $$ qva{l}_{j,h}= pva{l}_{j,h}\ast \frac{m}{r_{j,h}} $$

In this method *m* is described as maximum number of interactions between all regions.

### HiCNorm

HiCNorm was developed by Ming Hu et al.*.* HiCNorm assumes a Poisson distribution to model sequencing errors and artefacts. It normalizes Hi-C contact maps and estimate the bias effects by using the effective length feature and the GC content feature while fixing the mappability feature as a Poisson offset. In this process, the normalized Hi-C contact map (*e*) for chromosome *i* at locus *j* and *h* is calculated based on effective length feature (*x*), GC content feature (*y*), the mappability feature (*z*) and Hi-C contact map *u*. The equations for intra-chromosomal Hi-C interactions follow as:

5 $$ {e}_{j,h}^i=\frac{u_{j,h}^i}{t_{j,h}^i} $$

where *t* calculated by:

6 $$ {t}_{j,h}^i=\exp \left[{\beta}_0^i+{\beta}_{len}^i\lg \left({x}_j^i{x}_h^i\right)+{\beta}_{gc}^i\lg \left({y}_j^i{y}_h^i\right)+\lg \left({z}_j^i{z}_h^i\right)\right] $$

Equations for the intra-chromosomal Hi-C interactions between chromosomes *i*_1_ and *i*_2_ are:

7 $$ {e}_{j,h}^{i_1{i}_2}=\frac{u_{j,h}^{i_1{i}_2}}{t_{j,h}^{i_1{i}_2}} $$

where *t* is calculated by:

8 $$ {t}_{j,h}^{i_1{i}_2}=\exp \left[{\beta}_0^{i_1{i}_2}+{\beta}_{len}^{i_1{i}_2}\lg \left({x}_j^{i_1}{x}_h^{i_2}\right)+{\beta}_{gc}^{i_1{i}_2}\lg \left({y}_j^{i_1}{y}_h^{i_2}\right)+\lg \left({z}_j^{i_1}{z}_h^{i_2}\right)\right] $$

### Fit-Hi-C

The Fit-Hi-C method was developed by Ferhat Ay et al.. This method uses a binomial distribution and works on intra-chromosomal interactions. In the first step, this method assumes that a single observed contact is equally likely to come from any of the M possible pairs of loci, so the null probability of this contact being between a specific locus pair is *p* = 1/M. Therefore, the probability of a given pair that has an exactly k contact count is:

9 $$ \mathit{\Pr}\left(K=k\right)=\left(\begin{array}{l}N\\ {}k\end{array}\right){p}^k{\left(1-p\right)}^{N-k} $$

The *P*-value is the corresponding cumulative probability of observing at least k contacts is:

10 $$ P\left(K\ge k\right)=\sum \limits_{i=K}^N\mathit{\Pr}\left(\mathrm{K}=\mathrm{i}\right) $$

In the second step, this method replaces the binned binomial method (contact probability *p)* with a spline-fitting procedure that provides a more precise estimate of the probability of observing a contact with a specified genomic distance (*d*_*j*, *h*_). In other words, Fit-Hi-C replaces in Eq. (9) the contact probability p with a function f ^(1)^(d) which is computed by a spline fit to observe contact probabilities of locus pairs based on their genomic distances. To achieve a smooth spline fit, this method segregate the locus pairs into *b* equal-occupancy bins (in this method b = 200). The smallest distances in bin *i* and bin *i + 1* define the lower and upper genomic distance boundaries, *s*_*i*_ and *e*_*i*_, respectively, for bin *i*. Then, for each bin *i*, this method computes three values: (1) the average number of contact counts per locus pair (*c*_*i*_); (2) the prior contact probability that a given mid-range read comes from one specific locus pair in this bin $\frac{{\mathrm{c}}_{\mathrm{i}}}{\mathrm{N}}$, where *N* is the total number of mid-range reads; and (3) the average interaction distance *d*_*i*_ over all locus pairs in the bin, including pairs that have a contact count of zero. This method then fits a univariate spline to the resulting b points $\left(\left({d}_i,\frac{c_i}{\mathrm{N}}\right),\dots, \left(\ {d}_b,\frac{c_i}{\mathrm{N}}\right)\right)$.

11 $$ {f}^{(1)}(d)=\sum \limits_{i=K}^b{f}^{(1)}\left({d}_i\right){\mathrm{f}}_i(d) $$

In the third step, this method uses a two-phase spline fitting procedure to modify the binning method, which involves producing a more accurate estimate of the null distribution by excluding contacts that are likely to be real.

### Visualize Hi-C interactions

In MHiC, we developed a Graphical User Interface for MHiC. The Interface enables the user to set parameters, generate significant interactions and also visualize Hi-C contact maps. We implemented the visualization as a HTML page to show Hi-C interactions on an Arc diagram or a Heatmap. The MHiC user interface contains two sections: 1) call background models to detect statistically significant interactions; 2) visualization options to visualize a Hi-C contact map. You can find more details about parameters related to each section in the user manual supplementary file.

### Data

The visualization part of MHiC needs a file with five columns (chr1, locus1, chr2, locus2 and readCount) to create an Arc diagram or a Heatmap diagram.

### Arc diagram

The Arc diagram only works on cis interactions. The number of interacting reads between two regions impacts on the thickness of the Arc link. Users can change the data resolution without doing any process of changing data resolution. In addition, MHiC can visualize interactions in a specific range of fragments and also users can select each fragment and visualize that region’s interactions. In addition, users can easily set a threshold for read counts to remove interactions that has fewer read counts than the threshold. MHiC can import different annotation files to show on top of the Arc Diagram and it can show valid interactions by changing colors or removing invalid interactions. This is also noticeable that the user can change the diagram’s colors if they need it.

### Contact map Heatmap

The Contact map diagram draws a Heatmap of interactions. The Heatmap Diagram can show interactions within a single chromosome multiple chromosomes or whole genome. In this diagram, the number of interacting reads or *p*-value between two regions impacts on the interaction’s color. Users can select interactions color range so when a user selects yellow and black, the interaction read count will show in the range between these colors. Also, the contact map diagram has the same options as the Arc Diagram except it can show the whole genome or inter-interactions.

## Results

To examine the performance of MHiC, we applied MHiC on data from mouse embryonic stem cell sample from Dixon database. All the analysis was performed on a system with 8-Gigabyte ram and core i7 CPU.

**Fig. 2:**
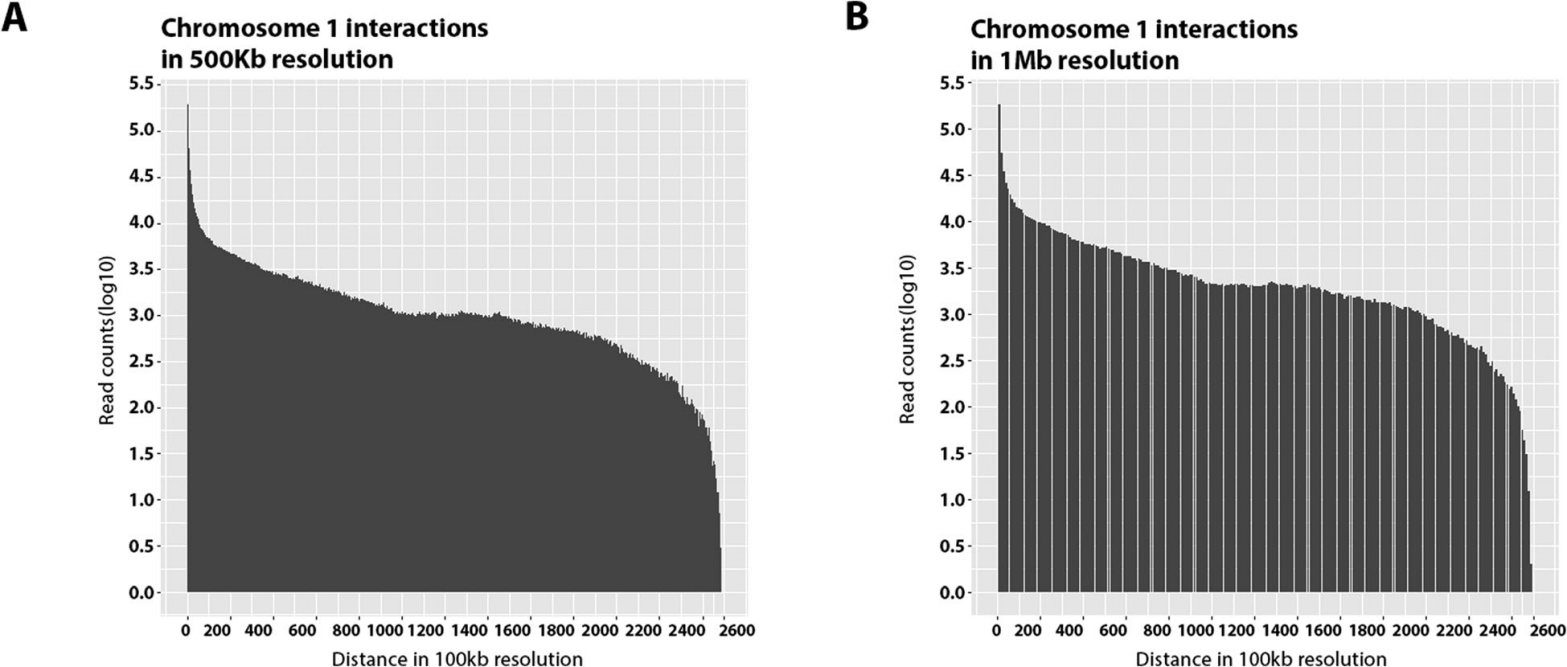
Database histogram. **a** Dixon Chromosome 1 interactions histogram in 500Kb resolution. **b** Dixon Chromosome 1 interactions histogram in 1 Mb resolution

First, we used HiC-Pro to generate Hi-C interactions and make the contact maps on bin-size 500Kb and 1 Mb. Figure 2 shows the database histogram, and Table 1 presents the statistical summary of HiC-Pro. As one can see in this table, HiC-Pro detected 41,006,364 interactions. We used these interactions as the input for MHiC.

**Table 1:** Dixon Database information after applying to HiC-Pack

Interaction Counts	Average read counts	bin-size	Total read counts	intra-chromosomal interactions	inter-chromosomal interactions
41,006,364	1.36	100 kb	56,168,196	5,225,136	35,781,228

**Fig. 3:**
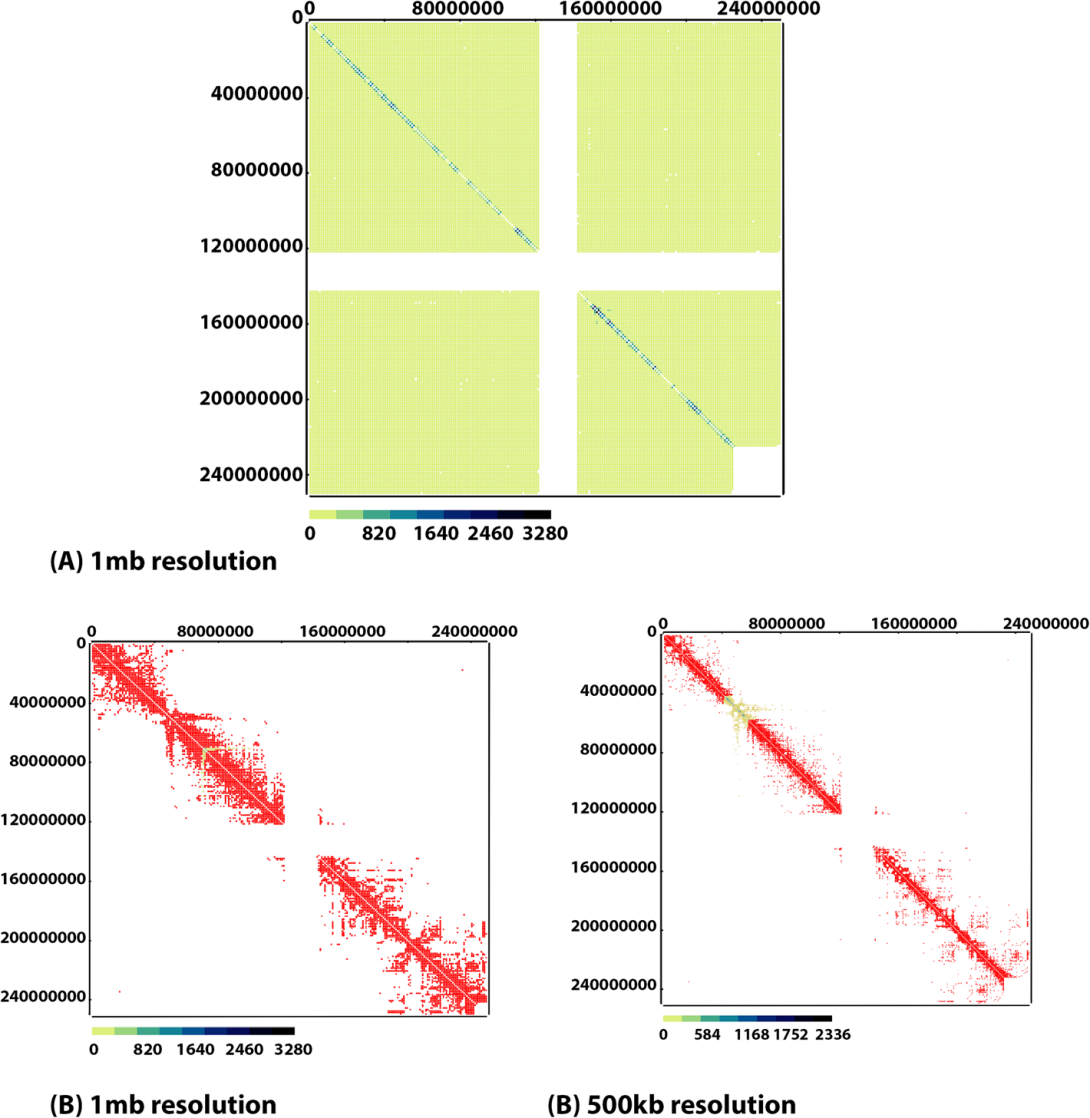
Hi-C interactions Heatmap diagram at 500Kb and 1 Mb resolutions for the entire Dixon chromosome 1, which was modeled by GOTHiC. **a** raw interactions contact map for chromosome 1 at 1 Mb resolution. **b** valid interactions contact map for chromosome 1 showed with red color for 500Kb and 1 Mb resolution

**Fig. 4:**
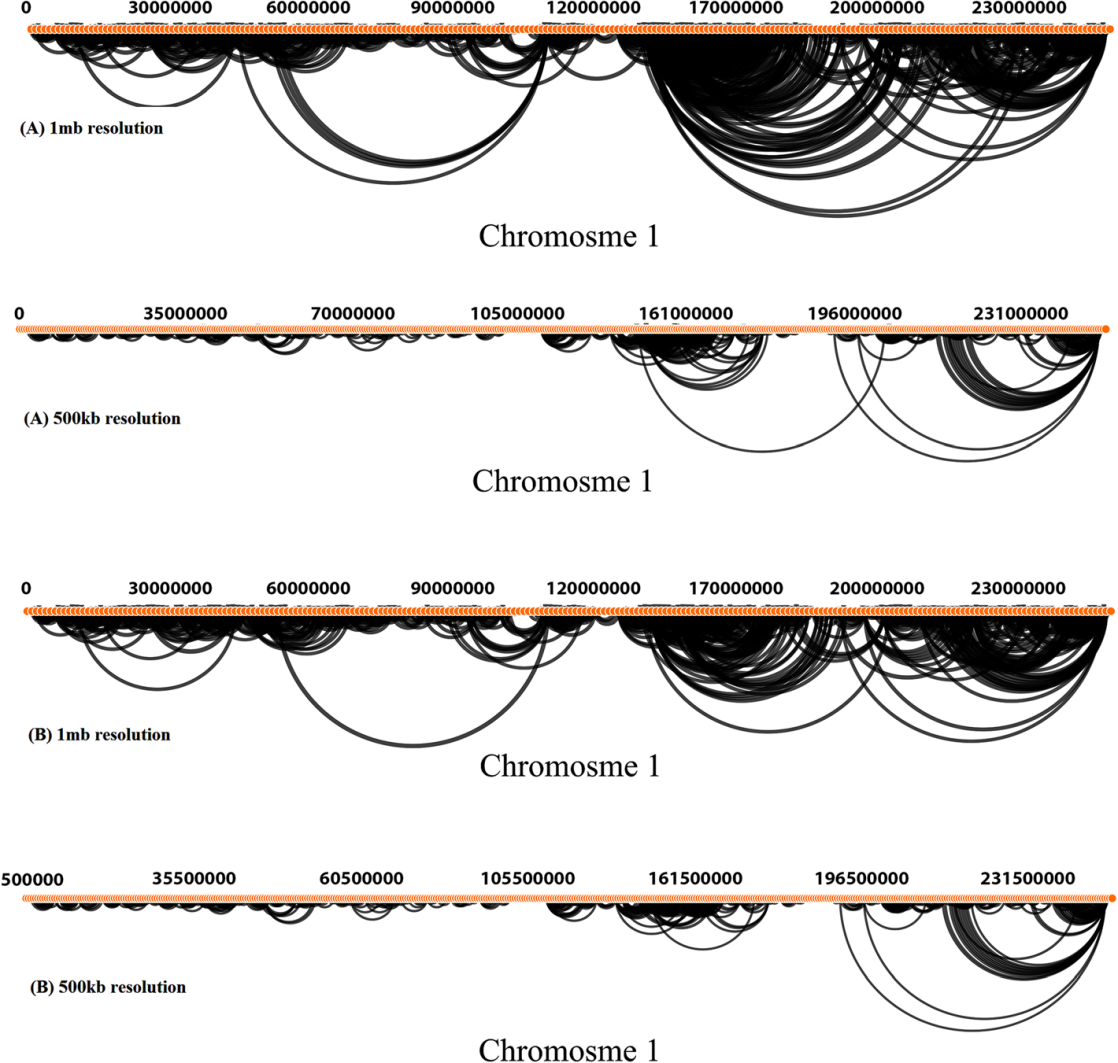
Hi-C interactions Arc diagram at 500Kb and 1 Mb resolutions for the entire Dixon chromosome 1, which was modeled by GOTHiC. **a** Arc diagrams to show interactions which have at least 50 read counts for 500Kb resolution and 100 read counts for 1 Mb resolution. **b** significant interactions’ Arc diagram

**Fig. 5:**
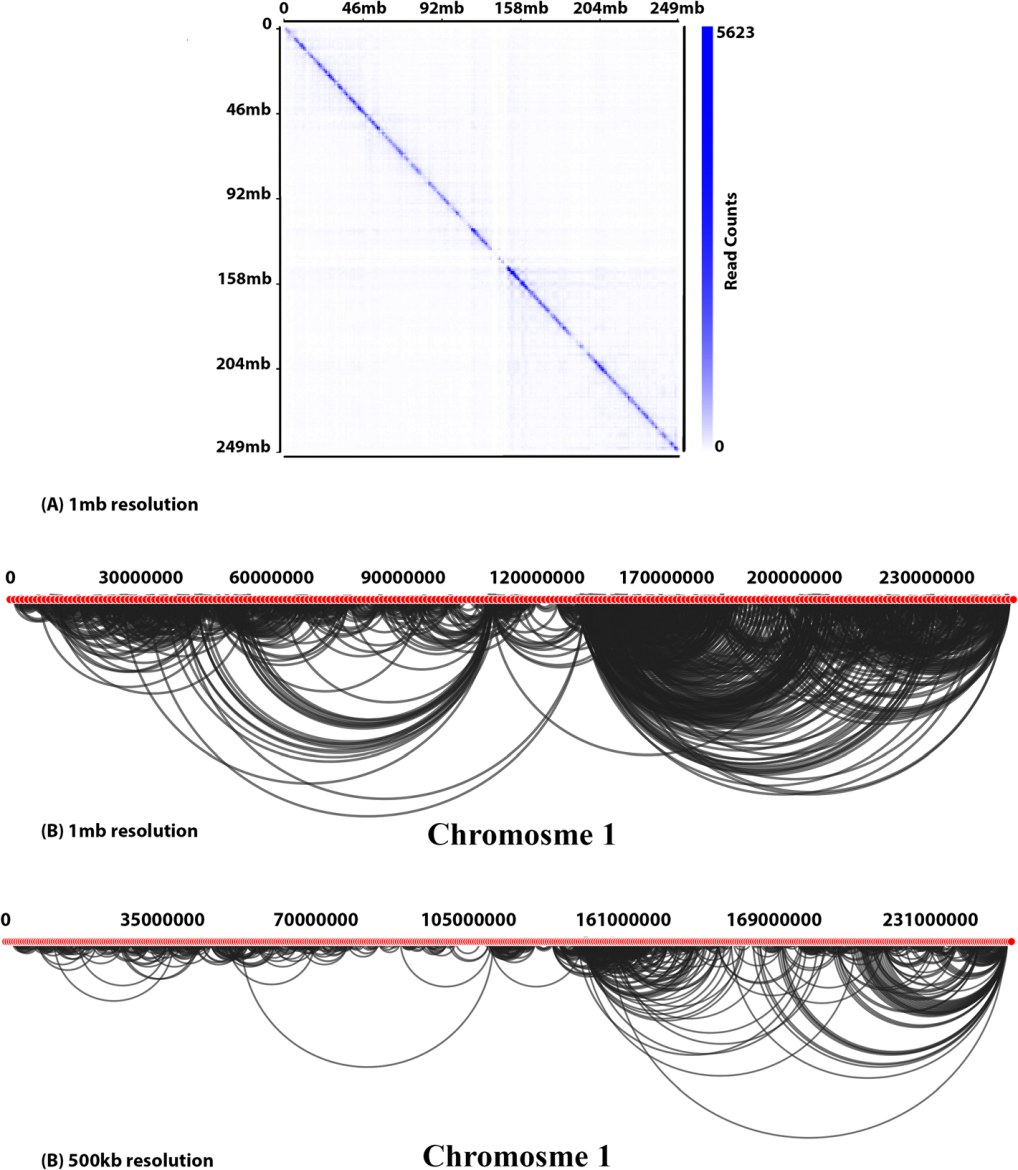
Hi-C interactions Heatmap diagram and Arc diagram at 500Kb and 1 Mb resolutions for the entire Dixon chromosome 1, which was modeled by Fit-Hi-C. **a** interactions contact map for chromosome 1. **b** Arc diagrams to show interactions which have at least 50 read counts for 500Kb resolution and 100 read counts for 1 Mb resolution

We run MHiC with two different settings (for GOTHiC and Fit-Hi-C methods) to create background models from the Dixon dataset chromosome 1. GOTHiC and Fit-Hi-C identified 5362 and 12,351 significant interactions on bin-size 500Kb and 3027 and 7062 significant interactions on bin-size 1 Mb. Also, for this dataset HiCNorm method need at least 12-Gigabyte ram to run smoothly. Therefore, we did not use this method outputs in results. These results are available in Table 2. Then, we used both raw Hi-C interactions and significant interactions identified by GOTHiC and Fit-Hi-C to visualize the interactions in both the Arc diagram and Heatmap plots (Figs. 3, 4 and 5).

**Table 2:** Dixon database Chromosome 1 information after applying HiC-Pack to MHiC at 500Kb and 1 Mb. In this table, the first row shows the number of interactions and average read counts before applying to MHiC. The GOTHiC and Fit-Hi-C rows show the number of significant interactions and its average read counts for each method

Methods	Interactions in 500Kb	Average read counts	Interactions in 1 Mb	Average read counts
Raw	Total: 98912	11.73	Total: 26302	40.49
GOTHiC	Significant: 5362	84.12	Significant: 3027	153.49
Fit-Hi-C	Significant: 12351	76.91	Significant: 7062	141.07

**Fig. 6:**
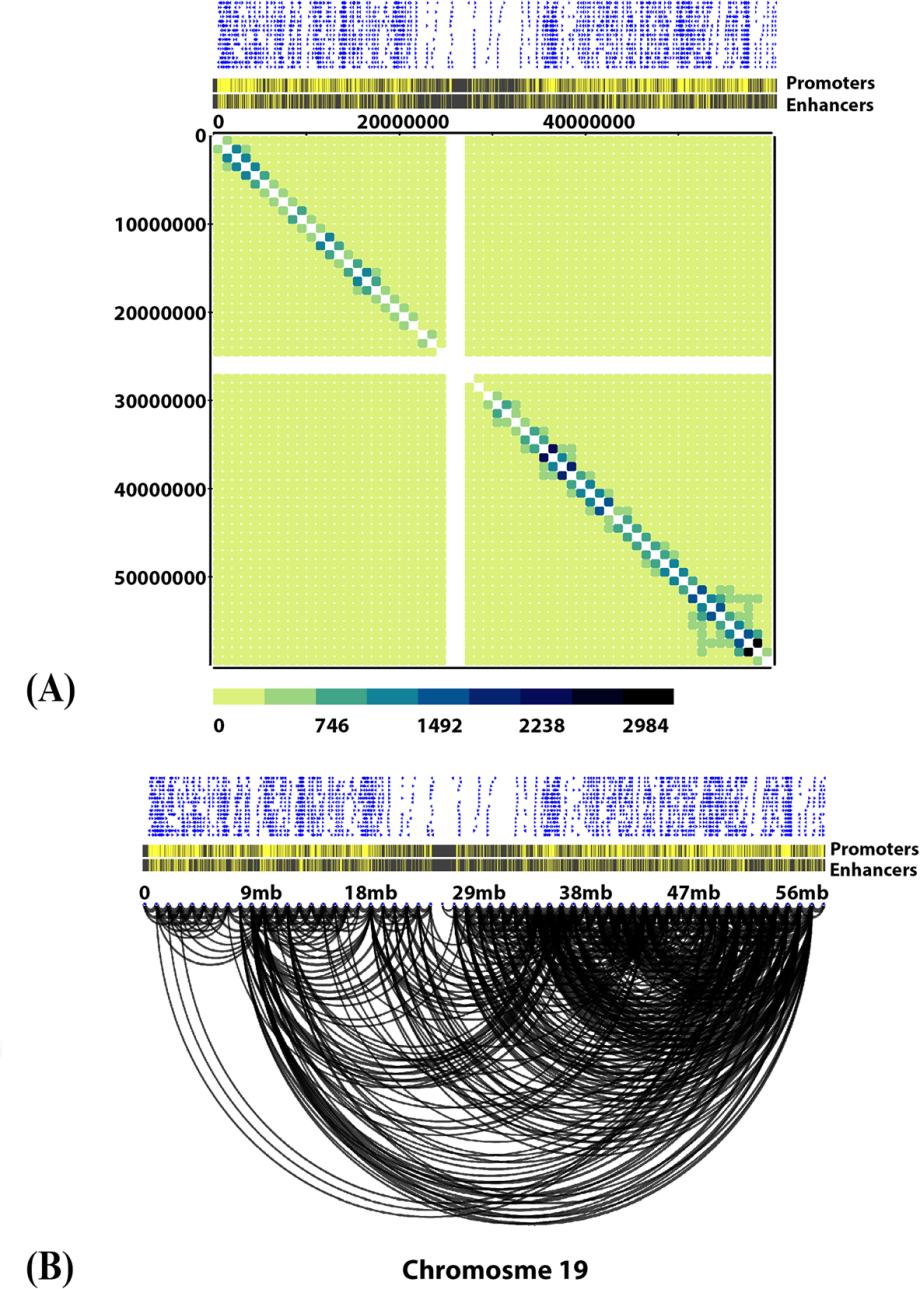
Hi-C interactions Heatmap diagram and Arc diagram with annotations at 1 Mb resolutions for the entire Dixon chromosome 19, which was modeled by GOTHiC. **a** raw interactions contact map. **b** Arc diagram to shows interactions that have at least 100 read counts with annotation

**Fig. 7:**
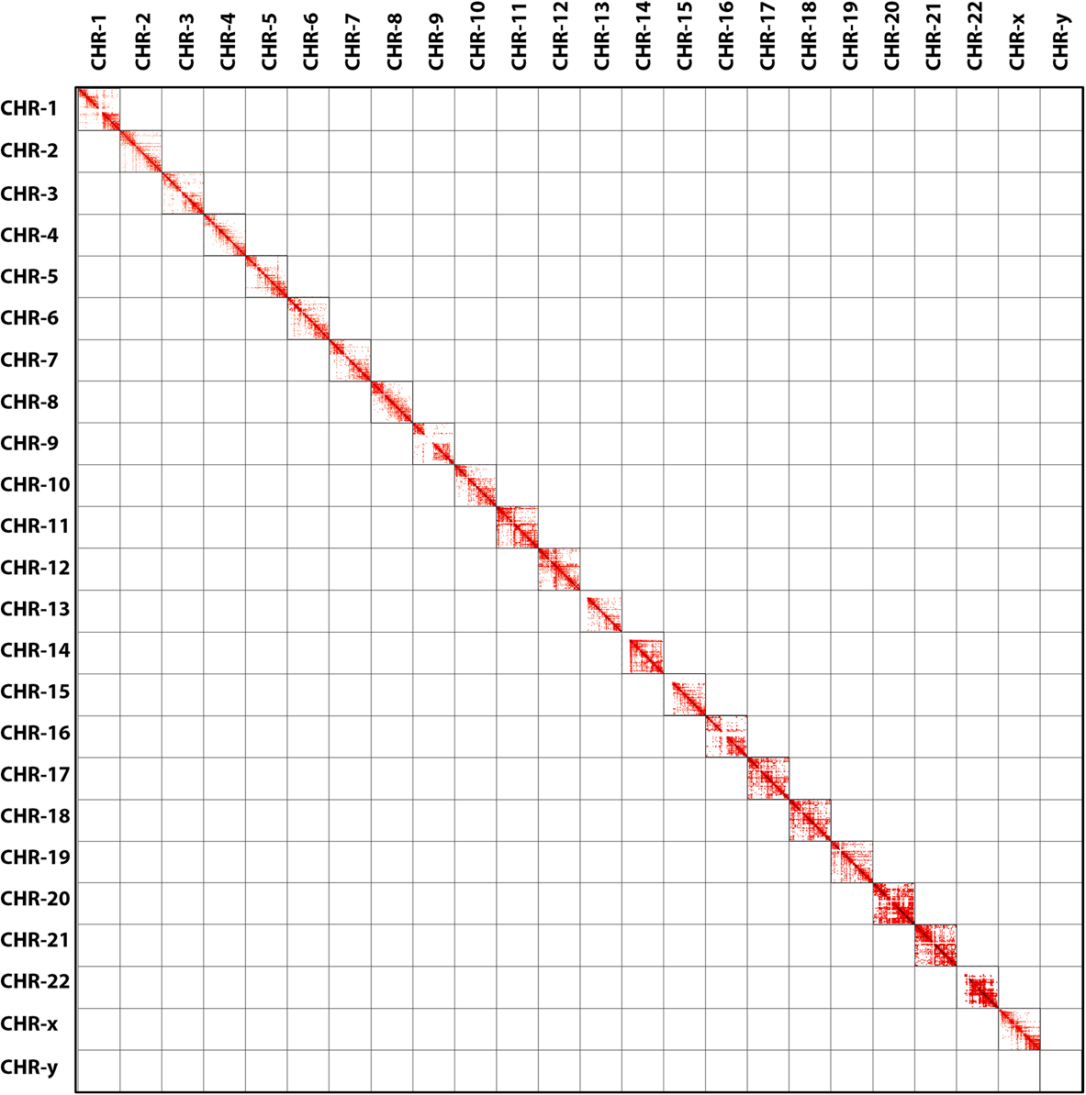
Hi-C interactions Heatmap diagram and Arc diagram with annotations at 1 Mb resolutions for the entire Dixon dataset. This diagram shows interactions that have at least 100 read counts

In the Arc diagrams the annotations are shown on the top. We used an option in MHiC to annotate Hi-C interactions by ENCODE predicted promoters and enhancers. Figure 6 shows enhancers and promoters coloured yellow and the promoters strand direction shown in blue. We also also generate a whole-genome contact map in 1 Mb bin-size from GOTHiC output which show interactions have at least 100 read counts (Fig. 7).

## Conclusions

In this article, we introduce MHiC. Currently MHiC has two main sections for modeling and visualizing Hi-C data. The tool is capable of receiving procced Hi-C data from different pipelines and identify statistically significant interactions by different Hi-C background models. MHiC also enables users to visualize interactions. The visualization section is able to create an Arc or Heatmap diagram from both the raw or the significant interactions.

## Availability and requirements

Project name: MHiC

Project home page: https://github.com/MHi-C/MHiC

Operating system(s): Platform independent

Programming language: R and JavaScript

Other requirements: R 3.6.1

License: GNU GPL.

Any restrictions to use by non-academics: License is not needed.

## Supplementary information

**Additional file 1.** MHiC classes and functions. Description of all functions and classes available in MHiC.

**Additional file 2.** MHiC user interface. Description of MHiC UI and MHiC visualization section.

## Data Availability

In this paper, we use mouse embryonic stem cell sample from Dixon database which can be accessed at http://promoter.bx.psu.edu/hi-c/download.html. Also, the MHiC source code available at https://github.com/MHi-C/MHiC. The HiC-Pro Outputs from Dixon dataset and MHiC user interface can be accessed at https://github.com/MHi-C/MHiCUI. For more details about each parameter, please visit the GitHub page of the project.

## Abbreviations

3C: Chromosome conformation capture
4C: Chromosome conformation capture-on-chip
5C: Chromosome conformation capture carbon copy
GC: Guanine-cytosine
Hi-C: High-throughput chromosome conformation capture
