# Supplementary material for: MHiC, an integrated user-friendly tool for the identification and visualization of significant interactions in Hi-C data
Source: BMC Genomics. 2020 Mar 12;21:225. doi: 10.1186/s12864-020-6636-7 (PMC7068949; doi:10.1186/s12864-020-6636-7)
Supplement: Supplementary file 1 — Additional file 1. MHiC classes and functions. Description of all functions and classes available in MHiC. [file 12864_2020_6636_MOESM1_ESM.docx]

**This documentation has three sections:**

1. **Overview**
2. **Description - functions and method used in this tool**
3. **User Guide – how to use this tool**
4. **Overview**

Hi-C is a powerful technique to understand genome organization. Unfortunately, Hi-C is biased so Hi-C data should be normalized in order to identify which interactions are significant. several tools that have been developed to normalize Hi-C data; these tools create different data structure from the raw Hi-C data. In addition, many methods that only worked with one or two data structures have been implemented to identify significant interactions.

In order to create an integrated tool, we developed a tool based on GOTHiC, HiCNorm, and Fit-Hi-C methods for identifying the significant interactions. Also, this tool gets the data from different tools such as HiCUP, HiC-Pro, and HOMER and creates a background model.

1. **Description**
   1. **Data**

This tool can identify the significant interactions from Hi-C data which is normalized by different tools such as HiC-Pro. In this part, we describe tools and data structures which are accepted by this tool.

The HiC-Pro output is a matrix file with three columns: Locus1 id, Locus2 id and Interaction counts (number of interacting read between two regions), and a bed file with four columns: chromosome ID, fragment start position, fragment end position, and fragment ID.


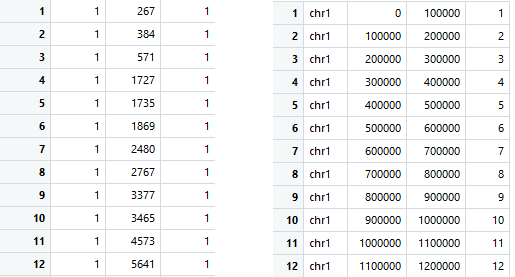


Figure 1 HiC-Pro outputs. Interactions file (matrix) on the left and bed file on the right

HiCUP outputs include two text files. A file with four columns: id; flag; chromosome; locus position, and a digest file which includes chromosome ID, fragment start position, and fragment end position. In the first file two separate rows with the same ID defines an interaction. In order to create this structure, users should use the hicup2gothic script which is available with the HiCUP tool.

HOMER processes FASTQ and bowtie2 files to map and perform quality control on Hi-C data. In this process, HOMER creates some CSV files to define Hi-C interactions for the next processing steps. In order to create this structure, users should visit the [HOMER](http://homer.ucsd.edu/) website.

- 1. **Method**

We developed this tool based on GOTHiC, HiCNorm, and Fit-Hi-C methods. Also, we used cumulative binomial tests to identify significant interactions between distal genomic loci that have signiﬁcantly more reads than expected by the chance in Hi-C experiments. Cumulative binomial tests are implemented based on GOTHiC and it is left as optional for users to apply it on other methods.

- 1. **Functions**

In this section we describe the main functions (main, binomial_function, MHiC, get_HiC_data, HiCnorm, FitHiC) and the parameters used in this tool.

- - 1. **MHiC:**

The main function of the tool takes Hi-C data from the user and gives back significant interactions for a given bin size.

**Usage**

MHiC (reads_file, Digest_file, sample_name, tools_name, res, cistrans , parallel, cores, removeDiagonal, save, remove, min_cov, min_len, min_gc, min_map, biasfile, noOfPasses, noOfBins, mappabilityThreshold, distUpThres, distLowThres)

**Within Arguments**

“reads_file”: Path of file which containing the read pairs information.

“Digest_file”: Path of digest file, which used to map reads (if users set tools_name “MHiC”, digest file no needed).

“sample_name”: A character string that will be used to name the output.

“tools_name”: A character string that gives the tool name that selects the method which is used in get_Hic_data functions.

“Res”: An integer that gives the required bin size (resolution) of processing.

“Cistrans”: A character string with three possibilities:
"all": runs methods on all interactions,
"cis": runs methods only on intrachromosomal/cis interactions,
"trans": runs methods only on interchromosomal/trans interactions.

“Parallel”: Logical argument. If TRUE the GOTHiC method will perform in parallel mode and multiple cores. This option is used to improve runtime.

“Cores”: An integer specifying the number of cores used in the parallel processing if parallel: TRUE.

“removeDiagonal”: Logical argument. If TRUE the diagonal interactions are removed and methods only apply on non-diagonal interaction.

“Save”: Logical argument. If this parameter is TRUE, the output saves in the default directory.

“Remove”: Logical argument. If this parameter is TRUE, the invalid interaction removes from the output.

“min_cov =2, min_len =0.1, min_gc =0.3, min_map =0.8”: Numeric values that define minimum coverage, length of interaction, gc, and mappability. These parameters are also required for HiCNorm.

“biasfile, noOfPasses =1, noOfBins =100, mappabilityThreshold =1, distUpThres =-1, distLowThres =-1”: Parameters that are required for Fit-Hi-C method. This is also noticeable that these parameters have default values.

- - 1. **get_Hic_data:**

The main import function, that takes read pairs, digest files with the tool’s name (required for how to read Hi-C data), and gives back a structured Hi-C matrix that is used in binomial_function, HiCnorm, and FitHiC functions.

- - 1. **Binomial, HiCnorm, and FitHiC functions:**

Main process functions, that get mapped interactions and apply it to named methods and gives back significant interactions for a given bin size.

1. **User Guide**
   1. **Requirements:**

Full functionality requires R (tested with version 3.4.2). It works on Ubuntu, macOS, and Windows (tested on Windows 10).

- 1. **Installation:**

MHiC is written in R. To install MHiC from github:

1. first install devtools package:

install.packages("devtools")

1. Load the devtools package

library(devtools)

1. use install_github("author/package").

install_github("Skhakmardan/MHiC")

- 1. **Example:**

We describe an example of using the MHiC tool.

Download MHiC_sample_data. Copy data folder in MHiC folder path.

1. Use MHiC function:

library(MHiC)

dirPath <- system.file("data","HiC-Pro", package="MHiC")

fileName1 <- list.files(dirPath, full.names=TRUE)[1]

fileName2 <- list.files(dirPath, full.names=TRUE)[2]

Output<-MHiC(fileName2, fileName1, "dixon_2M_100000", "HiC_PRO", res = 1000000, cistrans = "all", parallel=FALSE, cores=NULL, removeDiagonal=TRUE, save=TRUE, remove= false )

1. Use user interface to do these functions from a visual UI. This UI gets requirement parameters level by level.
