# Supplementary material for: MHiC, an integrated user-friendly tool for the identification and visualization of significant interactions in Hi-C data
Source: BMC Genomics. 2020 Mar 12;21:225. doi: 10.1186/s12864-020-6636-7 (PMC7068949; doi:10.1186/s12864-020-6636-7)

**This documentation has two sections:**

1. **Overview**
2. **User Guide – how to use this tool**
3. **Overview**

In order to have an integrated tool, we developed a Graphical User Interface for MHiC. This graphical User Interface enables the user to set parameters and generate significant interactions and also visualize a Hi-C contact map. We developed this part as an HTML page. In this document, we will describe each part of the user interface and diagrams page.

1. **User Guide**

We explain this section in two parts:

- 1. **User Interface**

We developed this user interface to provide an easier way for users’ operations with the analytical section of the tool. This page receives data and its options from users and sends it to the analytical section. In other words, we use the main section as a local server.


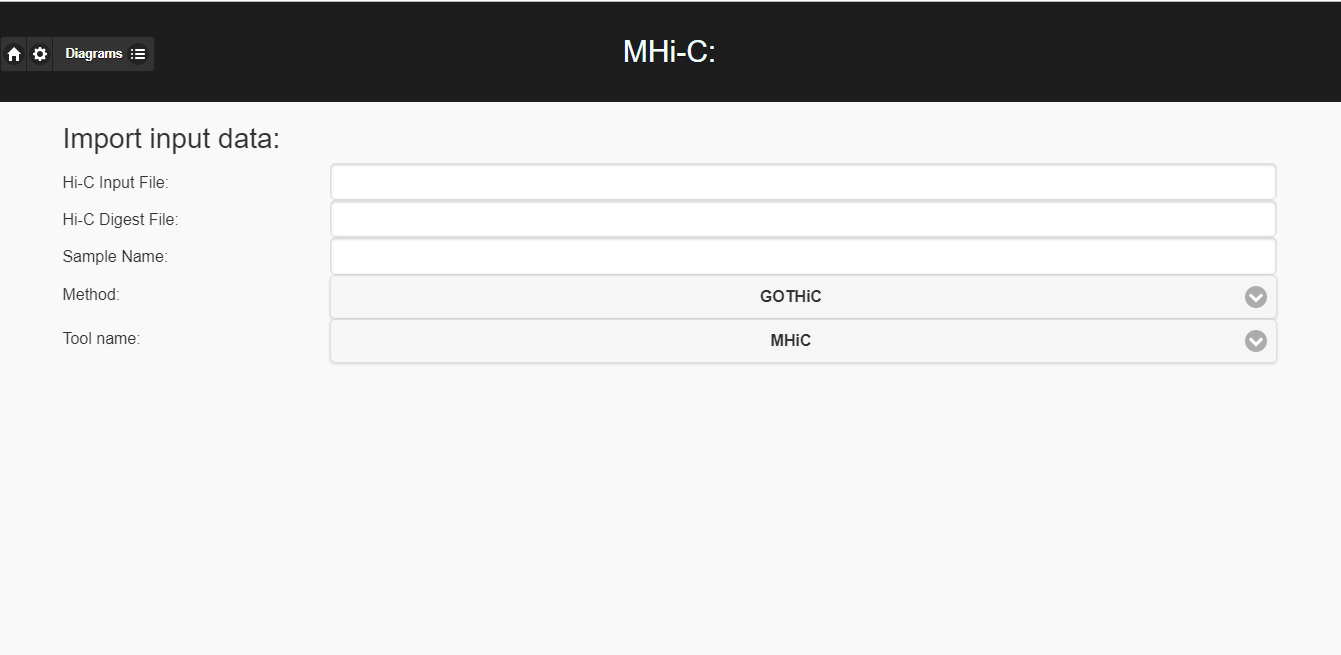


This HTML page contains two types of elements. In the center, we implemented the import data section to receive data from users and process it in R functions. This section includes:

“Hi-C Input File”: Path of a file that contains the reads (interactions) information. Instead of the file path a user can set the file name as input if the data is located in the MHiC’s data directory.

“Hi-C Digest File”: Path of digest file, which used to map reads.

“Sample Name”: The name used for the tool’s outputs.

“Method”: In this part, users select the method used for Hi-C data analysis.

“Tool Name”: Defines the preparing process used on Hi-C data.


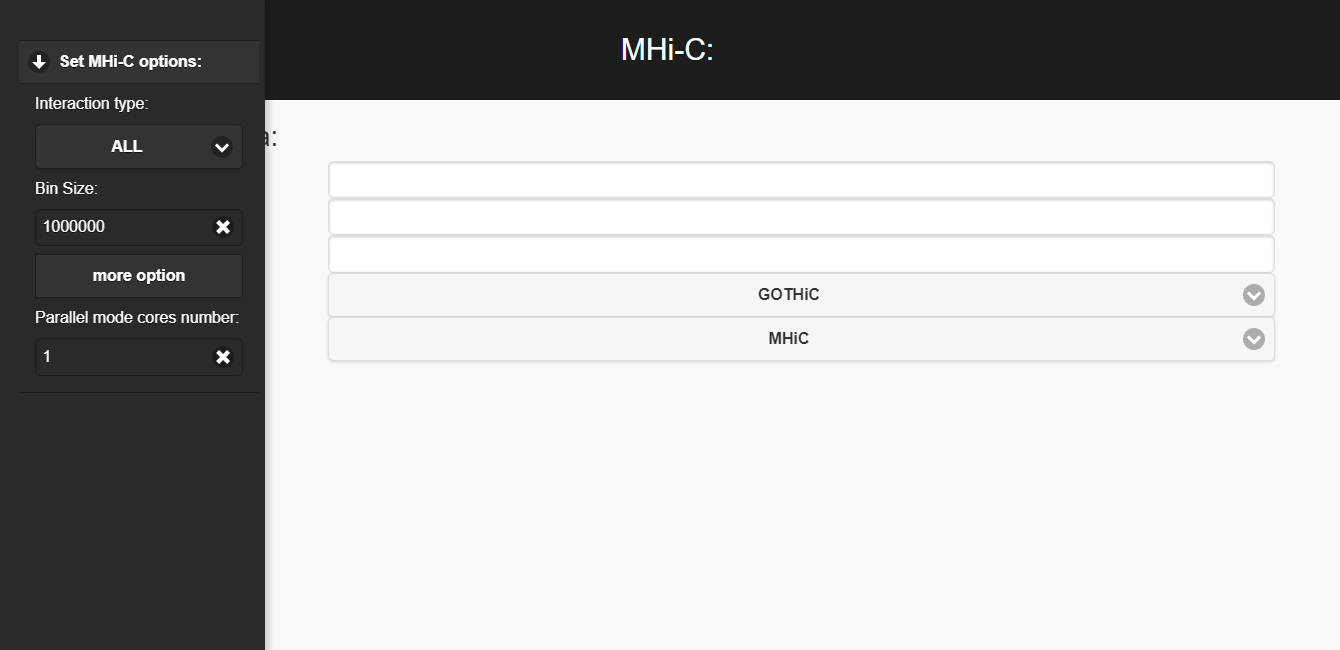


Along the import data section, we develop an options section on the left side to change the main process parameters. The option part contains six elements:

“Interactions type”: Specify that the R package process work on which type of interactions (inter or intra interactions).

“Bin size/resolution”: Sets the resolution of data in the process.

“Parallel mode (more options)”: Changes the process mode from single-core to multi-core.

“Remove diagonal interactions (more options)”: Removes interactions, that both sides of interactions have a same locus.

“Remove invalid interactions (more options)”: Removes interactions that are not significant interactions (at the process end).

“Core numbers”: Defines the number of cores for parallel processing.

- 1. **Visualizations**

In MHiC, we develop a visualization section. This section is implemented as HTML pages to show Hi-C interactions as either an Arc diagram or a Heatmap diagram.

**Arc Diagram**

Arc Diagram only works on cis interactions and use circular nodes to show locus positions. An Arc links between two nodes for each interaction. The thickness of the links results from the number of interacting reads. There are options to change node and link colours.


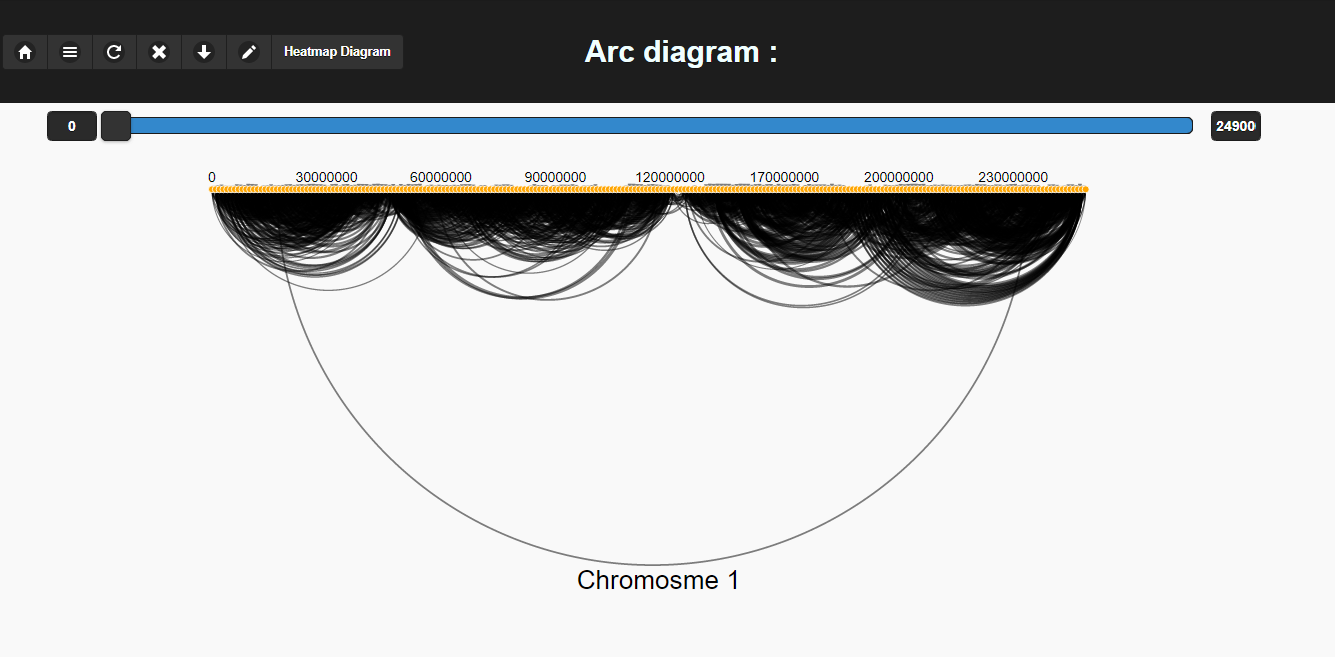


Users can select a particular node (locus position) or link (interaction between two regions) to show pieces of information about that locus or interaction.


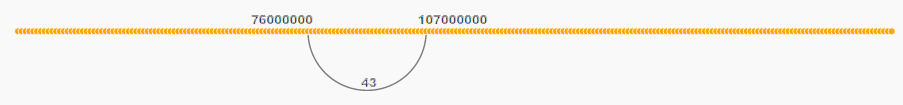


The other option that we provided is zoom in. This option allows a user to show interactions and loci between two particular positions.


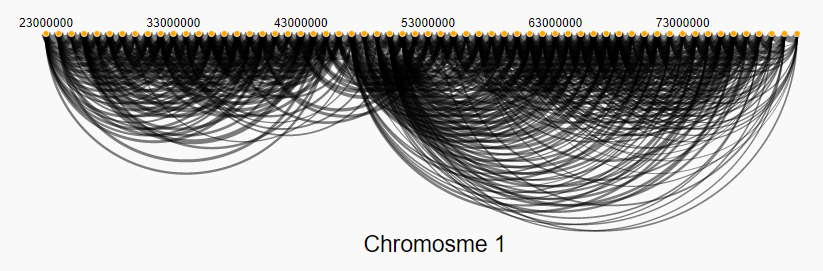


Along with the zoom in, we developed an option to set read counts and visualize Hi-C interactions that only have more read counts than the option value.

The other main element is the annotation files’ visualization. In this part, users can import different annotation files to show on top of the Arc diagram.

**Contact map diagram**


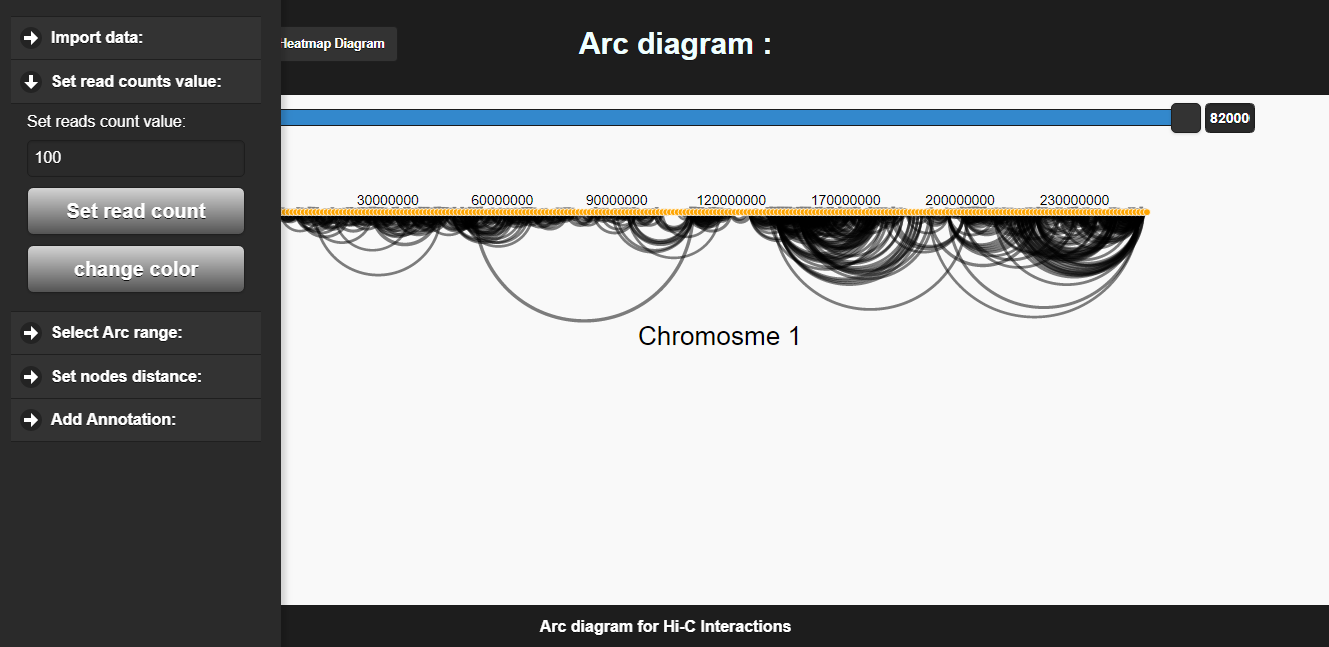


The contact map diagram shows a Heatmap of interactions. The Heatmap can show interactions within a single chromosome or multiple chromosomes. As well as Arc Diagram, users can import different annotation files to show on top of the Arc diagram. Also, users can set read counts, so interactions that have more read counts value visualizes in red or aother color that user selects.


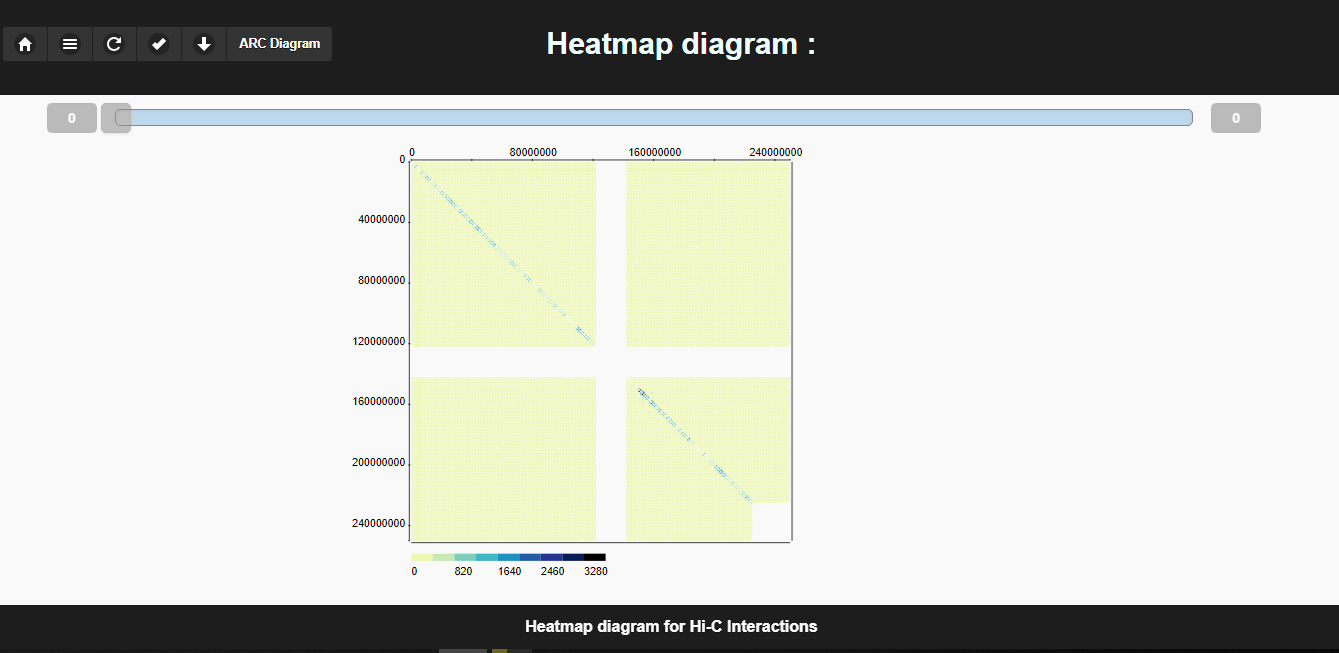

Supplement: Supplementary file 2 — Additional file 2. MHiC user interface. Description of MHiC UI and MHiC visualization section. [file 12864_2020_6636_MOESM2_ESM.docx]
